# Turning Copper and Aluminum Alloys with Natural Rocks as Cutting Tools

**DOI:** 10.3390/ma15062187

**Published:** 2022-03-16

**Authors:** Bernd Breidenstein, Berend Denkena, Benjamin Bergmann, Philipp Wolters, Tobias Picker

**Affiliations:** Institute of Production Engineering and Machine Tools, Leibniz University Hannover, An der Universität 2, 30823 Garbsen, Germany; breidenstein@ifw.uni-hannover.de (B.B.); denkena@ifw.uni-hannover.de (B.D.); bergmann@ifw.uni-hannover.de (B.B.); wolters@ifw.uni-hannover.de (P.W.)

**Keywords:** natural rocks, cutting tools, grinding, turning

## Abstract

The need for rare resources, such as tungsten or cobalt, combined with the high energy requirements to produce cutting materials, is forcing research and development to work out environmentally friendly alternatives. Natural rocks could be an alternative since they are available in large quantities worldwide, have a potentially suitable property profile, and do not require energy-intensive processes to make them usable as cutting materials. However, there are only a few studies on the usability and suitability of natural rocks as cutting materials for machining processes. Therefore, in this article, inserts made of natural rocks were ground and used in turning operations. First, the properties of various natural rocks were determined, as were the tool properties after grinding. Then, the tool load and wear during the machining process were recorded and evaluated to assess the potential applications of this alternative cutting material more accurately. It is therefore becoming apparent that flint and quartz are suitable for use as alternative cutting materials and should be further researched.

## 1. Introduction

High levels of productivity and resource efficiency are key factors for modern manufacturing processes, which is why high demands are made with regard to these factors. Therefore, constant improvement and redevelopment of cutting tools and process management are needed to meet these demands. One possibility is achieving higher material removal rates with lower cutting tool material costs. The need for rare raw materials and large amounts of energy for the production of modern cutting tool materials is a disadvantage of current high-performance cutting tool materials, such as polycrystalline diamond and cemented carbide [1]. Additionally, the availability of rare raw materials, such as tungsten or cobalt, can be critical, which will lead to additional challenges in the process chain of manufacturing. This is particularly the case for regions that have to import these materials to produce high-performance cutting tools, such as the United States [2] or the EU [3]. For this reason, research projects are looking for ways to replace these critical raw materials [4] or to avoid the use of these raw materials by using alternative cutting tool materials [5]. In this context, natural rocks could act as alternative cutting tool materials. The high number of easily accessible deposits available worldwide ensures that rocks have a high level of global availability. Since this potential cutting tool material is already available in nature in the required state, it is also possible to dispense with costly and energy-intensive preparation and manufacturing processes for the production of the cutting tool material. Using natural rocks as cutting tool material would, therefore, mean that, compared with the production of a kilogram of conventional cemented carbide, up to 480 MJ-equivalents of energy can be saved and emissions of climate-affecting gases can be reduced by up to 19 kg CO_2_-equivalents [1]. For these reasons, natural rocks could provide environmentally friendly alternative cutting tool materials to the tool industry and thus a potential alternative to conventional cutting tool materials. At the same time, this would provide the industry with the opportunity to advance the transition to a more sustainable economy, which is particularly important in the context of climate change. The machining of comparatively soft materials, such as aluminum and copper alloys or plastics, has already been identified as the first potential area of application in which natural rocks could be used as alternative, and environmentally friendly, cutting tool materials [6,7]. However, their operational behaviors, such as their process forces, tool wear and their interactions, have not been investigated so far.

One reason for these gaps in the state of the art is that research projects in the field of engineering sciences tend to deal with mining or civil engineering issues related to rocks. In the field of production engineering, for example, the investigation of the operational behavior of rock-cutting tools, such as cutting disks [8] or wire sawing tools [9], and the development of such tools is a frequent topic of research projects in this area. In contrast, a broader knowledge base is available regarding the mechanisms of material removal during the machining of rocks. Based on findings from scratch tests, it is assumed in the literature that rock stiffness [10], cutting depth [11], and the stress state at the cutting edge [12] are decisive factors for the material removal mechanisms that occur during machining. Investigations dealing with material failure mechanisms and the development of analytical models for process forces are also covered in the literature [13] and contribute to the understanding of rock machining. In addition, the formation process of the rocks and their chemical and structural composition influence their properties. The grain size and shape of the rocks, their texture and defects like cracks and pores also influence their properties [14]. Despite the existence of proposals for a standardized determination and description of rock properties in the literature, a wide range of indices and methods are used for this purpose. Furthermore, it is known that the transferability of the used methods to other use cases may not necessarily be possible [15].

Therefore, previous work has investigated the suitability of natural rocks as cutting tool material. Bending strength and texture orientation were defined as the main selection criteria for their usage as a cutting tool. The possibility of grinding indexable inserts from rocks and using them in turning operations was also demonstrated [6,7]. The present study extends these investigations to include the influence of tool and rock properties after grinding on the process forces during machining. With additional analyses of the tool wear, it is possible to determine process parameters for natural rock as cutting material.

## 2. Materials and Methods

### 2.1. Fabrication and Characterization of Cutting Tools Made of Natural Rocks

The five rocks used in this investigation were Alta quartzite, flint, lamellar obsidian, quartz, and silver quartzite. A rock saw (DEMA WB 2000, DEMA, Übrigshausen, Germany) and a cut-off grinding machine (Struers Discotom-10, Struers, Willich, Germany) were used to cut the raw rocks into the required sample geometry. Before cutting, the raw rocks were available as blocks or nodules with lengths between 80 and 250 mm, or as slabs with a thickness of up to 22 mm. The raw rocks were cut into samples with dimensions of 18 × 18 × 5.5 mm. To account for the influence of the existing mica textures in the quartzites on the material properties and operating behavior, as described in [6], the perpendicular orientation of the mica textures to the loading direction was ensured during the three-point bending tests and cutting tests. Afterward, the targeted sample thickness of 4.76 mm was acquired with a grinding process performed on a Blohm Profimat MC 407 (Blohm, Hamburg, Germany) grinding machine with a diamond grinding tool (D46, C100) with a metallic bond. The process parameters of this two-stage grinding process were a feed velocity of 3200 mm/min and a depth of cut of 20 µm for rough machining, and a feed velocity of 200 mm/min and a depth of cut of 5 µm for finishing. Finishing took place at a sample thickness of 4.78 mm. Both grinding steps were performed with a cutting speed of 30 m/s.

**Figure 1:**
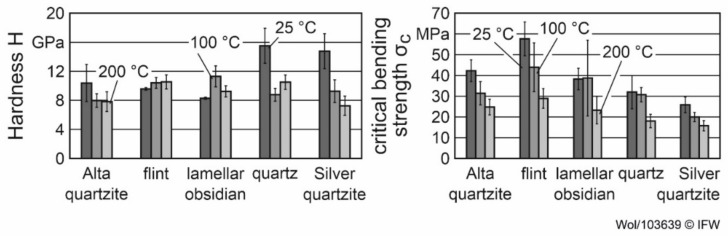
Hardness and critical bending strength of the rock samples at different temperatures.

The hardness H and the critical bending strength σ_c_ of the rock samples were used for the characterization of their mechanical properties. The critical bending strength can be used as a measure of load-bearing capacity and structural cohesion for solids with multiple phases, as has already been shown in [6,16]. The mentioned properties of the rocks used in this paper have already been investigated and discussed for different temperature levels in [17]. Therefore, the Vickers hardness (given in GPa) and critical bending strength of the rocks are shown at three temperature levels (25 °C, 100 °C, 200 °C) in Figure 1, as given in [17]. To determine the hardness of the rocks, five measurements were made at each temperature level on three samples of each rock. The critical bending strength was measured at each temperature level on five samples of each rock [17]. The values given in Figure 1 are therefore the mean values of these measurements. The hardness of all rocks was between 8 and 16 GPa for all rocks at 25 °C (see Figure 1). Consequently, all rocks were significantly harder than the aluminum (0.98 and 0.52 GPa) and copper alloys (0.67 and 0.93 GPa) to be machined.

Grinding of the indexable inserts was performed on a Wendt WAC 715 Centro (Wendt, Meerbusch, Germany) cutting insert grinding machine, as a plunge face grinding operation. A cup grinding wheel with a diameter of 400 mm, a width of the abrasive layer of 12 mm, a metallic bond, and a diamond as the abrasive (D46, C75) was used. A silicon carbide cup dressing roller with a grain size of d_g_ = 125 µm (ANSI #120) and a vitrified bond was used to perform a continuous dressing process in a counter direction parallel to the face of the dressing roller. A cutting speed of 2 m/s and a depth of cut of 0.5 µm per feed pulse was used in the dressing process. The grinding of the inserts was performed with a cutting speed of 22 m/s and a feed velocity of 29 mm/min. A low-viscosity mineral oil (R-Oil HM7, Rhenus Lub, Mönchengladbach, Germany) was used as a cutting fluid. Inserts of the type SNMN120404 were manufactured according to ISO 1832.

The surface roughnesses of the inserts after the grinding process were measured with a Confovis Duo Vario confocal microscope (Confovis, Jena, Germany) and analyzed with the software MountainsMap^®^ (Digital Surf, Besancon, France). The cutting edge roughness of the inserts and their cutting edge geometry were measured with an Alicona Infinite Focus G5 (Alicona, Raaba/Graz, Austria) focus variation microscope. Three samples of each rock were measured to determine the roughness values.

### 2.2. Turning Operations

**Figure 2:**
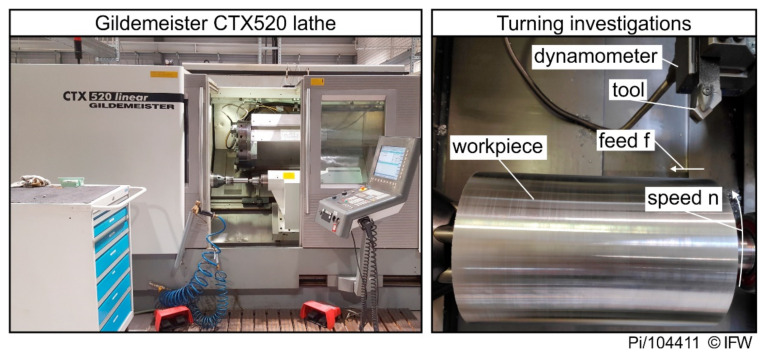
Experimental setup for the turning investigations.

All turning experiments were conducted with a Gildemeister CTX 520 (Gildemeister, Bielefeld, Germany) lathe were are repeated twice. A DSSNL2020K12 tool holder (Walter AG, Tübingen, Germany) was used to clamp the indexable inserts in the lathe. The holder led to a clearance angle of α = 6° and a rake angle of γ = −6° in the process (see Figure 2). All turning tests were performed without cooling with a constant depth of cut of a_p_ = 0.5 mm. The cutting speed v_c_ and the feed rate f were varied on two levels, v_c_ = 300/600 m/min and f = 0.1/0.2 mm, respectively. Cemented carbide inserts (SNMN120408) were used as a comparison cutting material to indicate the performance of the inserts made of rock. The composition was 94% tungsten carbide with 6% cobalt as the binder phase, where the average grain size of tungsten carbide is 0.6 µm (hardness 1900 HV30, fracture toughness K_IC_ = 9.0 MPa·m^1/2^, flexural strength = 3900 MPa). The cemented carbide inserts were coated with an 18 µm thick TiCN coating using a CVD coating process.

Two aluminum (Al2007T4 and Al5754T4) and two copper (CC496K and CW614N) alloys were chosen as workpiece materials. The material properties are shown in Table 1. By varying the material properties of the workpiece, the influence of different hardnesses, tensile strengths, or yield strengths can be determined. This allows further estimation of the application area of natural rocks as a cutting material.

Tool wear was inspected after a maximum feed path of L_f_ = 200 m, using a Keyence VHX5000 digital microscope (Keyence, Neu-Isenburg, Germany). The process forces were measured using a Kistler three-component dynamometer type 9121A. All investigations were repeated once.

## 3. Results and Discussion

### 3.1. Surface Roughness and Cutting Edge Properties of the Rock Inserts

**Figure 3:**
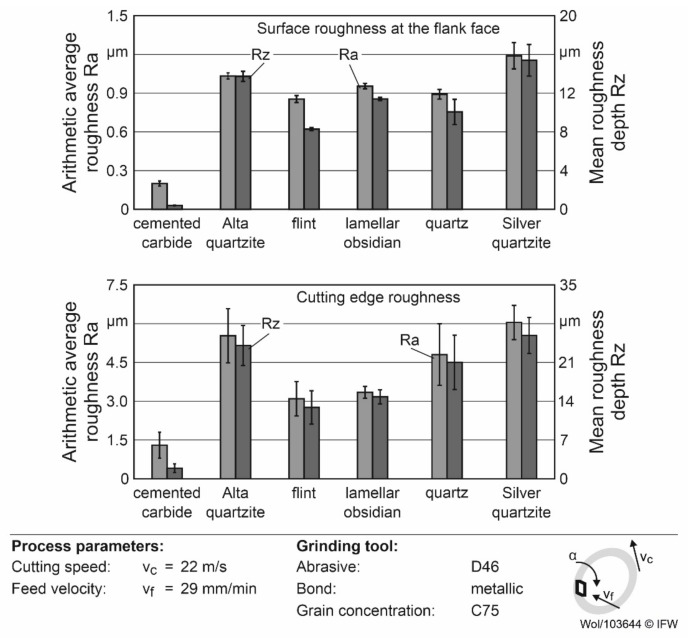
Surface and cutting edge roughnesses of the indexable inserts.

The results of the surfaces and cutting edge roughnesses after grinding are shown in Figure 3. The results show an arithmetic average roughness Ra between 0.85 and 1.19 µm and a mean roughness depth of Rz between 8.28 and 15.4 µm at the flank face of the rock inserts. Rocks with a higher critical bending strength tend to show a lower roughness at the flank face, as described in [6]. However, other factors, such as cracks in the microstructure and the interlocking of mineral grains, can also influence the roughness of the inserts after grinding [6], which is an explanation for the higher roughness values of Alta-quartzite in comparison to quartz, despite the higher critical bending strength of Alta quartzite.

The roughness of the cutting edge of the inserts was between Ra = 3.09 and 6.04 µm, while Rz was between 12.87 and 25.87 µm. The results showed a tendency towards an increase in cutting edge roughness with a decrease in critical bending strength. A decrease in the critical bending strength leads to a lower load-bearing capacity of the microstructure and can therefore facilitate the breakout of grains or material from the cutting edge due to the loads of the grinding process. This, in turn, leads to an increase in the roughness of the cutting edge. However, in this case, it cannot be ruled out that other factors played an important role in this context, as can be seen by a comparison of Alta quartzite and the quartz. In addition to the cracks and the interlocking of grains already mentioned, the stress state and grain size of the rocks were possible influencing factors. A tensile stress state near the cutting edge can, for example, influence the cutting edge roughness produced during grinding by facilitating crack propagation and brittle material removal through chipping, which in turn leads to an increase in cutting edge roughness compared to ductile removal. Considering the often-high grain size of rocks in comparison to technical materials, the breakout of a single grain or parts of grains as a result of the process loads in grinding can therefore increase the cutting edge roughness significantly.

In addition to the roughness of the inserts, the cutting edge segments at the flank face and the rake face, S_α,_ S_γ_ and their average cutting edge rounding $\;\bar{S}$, were determined according to [18] (see Table 2). It can be seen that the cutting edge rounding of the inserts differed depending on the rock investigated, and was between two and four times higher than for the cemented carbide inserts. The values of $\;\bar{S}$ showed a tendency comparable to the cutting edge roughness. An increase in cutting edge roughness can be accompanied by a change in the cutting edge geometry and thus alter the cutting edge rounding, as it can be linked to brittle material breakouts at the cutting edge. Brittle material removal counteracts the formation of a tapered cutting wedge due to the associated chipping and thus leads to the formation of a wider, more rounded cutting edge. This, in turn, leads to an increase of $\;\bar{S}$. Moreover, the material removal behavior at the rake and the flank face of the rocks can differ in the grinding processes resulting from the different load situations at these surfaces in the process and the different material properties of the rocks. This can influence the cutting edge geometry by different proportions of material removed from the faces, e.g., if the material removal at the flank face is dominated by ductile material removal and at the rake face is dominated by brittle material removal. As a result, the length of the cutting edge segments at the flank and at the rake face and the geometry of the cutting edge can be affected. It is, therefore, possible that the effects influencing cutting edge roughness were also important for the formation of the cutting edge geometry in the grinding process. However, more specific investigations are needed to verify this hypothesis, as this aspect is not the focus of this paper.

However, it must be mentioned that it is already known in the literature that the cutting edge geometry influences the operational behavior of cutting tools, e.g., by influencing the material flow in the separation zone or the mechanical and thermal loads [18,19,20]. It is therefore possible that the different cutting edge geometries of the inserts led to differences in their operational behavior. This could be especially true for the wear behavior of the inserts, as the mentioned differences in cutting edge roughness can be associated with brittle material removal, which is often linked with cracks in the microstructure. These cracks can facilitate wear through further brittle outbreaks if the process loads in turning are applied to the inserts in the turning process.

### 3.2. Application Area of Natural Rocks as Cutting Material

**Figure 4:**
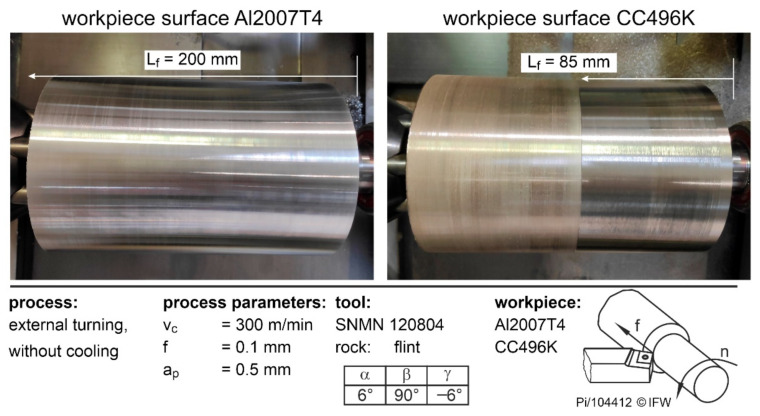
Workpiece surfaces and feed paths from cutting different workpieces with flint.

In order to be able to assess the potential of natural rocks as cutting materials for metalworking more accurately, it is first necessary to determine process parameters and workpiece materials for which no direct cutting edge failure occurs. For this reason, external turning tests were carried out by varying the cutting speed, the feed rate, the workpiece material, and the natural rock. The parameter combinations for which the tools can withstand a complete path length of 200 mm without breaking were checked. Whether the cutting edges of the tools broke during the path length could be identified in the form of heels on the workpieces. One example is shown in Figure 4, in which the workpieces Al2007T4 and CC496K are shown after machining with flint under v_c_ = 300 m/min and f = 0.1 mm. If this natural rock is used for aluminum, the cutting edge withstands the loads and can complete a feed path of L_f_ = 200 mm. If, on the other hand, copper is machined, the cutting edge breaks at a feed path of approximately 85 mm due to the high loads and the resulting wear.

**Figure 5:**
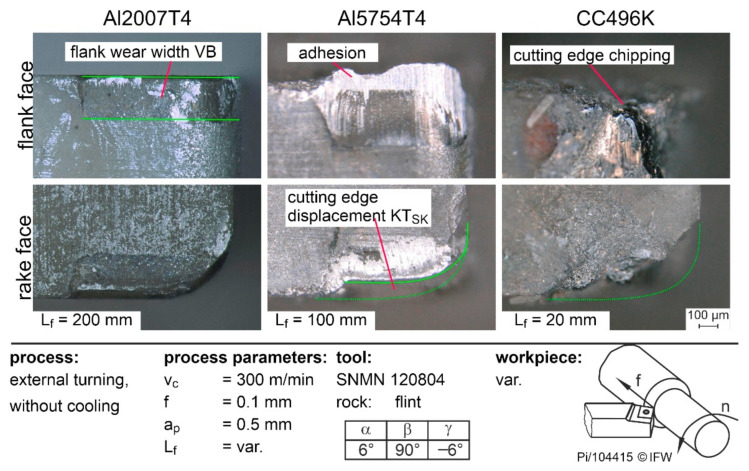
Tool wear using flint as cutting material.

Figure 5 shows the resulting tool wear when using flint for the three workpiece materials Al2007T4, Al5754T4, and CC496K. From the microscopic pictures, the different wear mechanisms can be easily identified. For instance, significantly abrasive wear in the form of the flank wear-width VB occurred during the machining of Al2007T4. When the softer Al5754T4 was machined, strong adhesions and the formation of a built-up edge occurred. In addition, the cutting edge is gradually torn out by the adhesive wear, resulting in cutting edge displacement. When CC496K was machined, the tool stresses exceeded the strength of the cutting material and the entire cutting edge broke.

**Figure 6:**
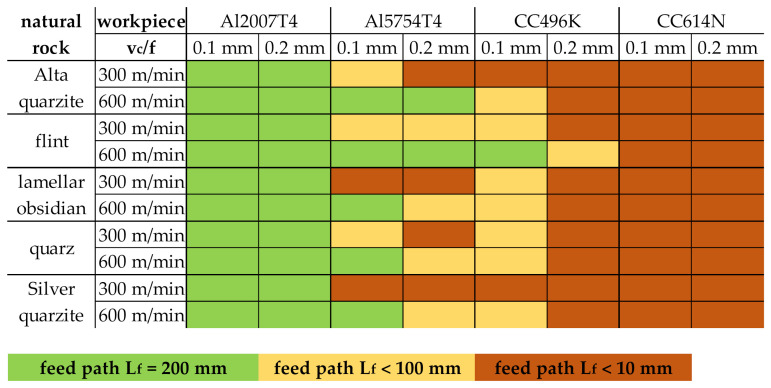
Comparison of the possible feed path without cutting edge displacement or chipping of all turning investigations.

An overview of the achieved feed paths as a function of the parameter variations is shown in Figure 6. It is plain to see that high mechanical loads as a result of the higher feed f = 0.2 mm at a low cutting speed v_c_ = 300 m/min led to increased breakage of the rock inserts. This finding can be transferred to all examined natural rocks. Therefore, high cutting speeds at low feed rates should be preferred when using rocks as cutting tools. In addition to the process parameters, the material properties also had a significant effect on the achievable feed path. For example, C614N could be machined with any of the five rocks, regardless of the respective process parameters. This could be attributed to the high Young’s modulus and yield strength, which require a high shear stress to cut. This exceeded the bending strength of the natural rocks, causing the cutting edges to break during machining. In contrast, the two aluminum alloys could be machined with all the stones, especially with a high cutting speed at low feed rates. However, when machining the softer Al5754T4, low cutting speeds led to a shortening of the feed path because the cutting edge roughness of the inserts combined with the low tensile strength led to severe adhesive wear of the cutting edge. Due to the adhesions, the edges were subsequently torn out (see Figure 5).

When comparing the application of the different rocks, it is noticeable that flint, with the highest critical bending strength σ_c_, can be used for most combinations over the entire path length. Even the copper alloy CC496K can be machined at high cutting speeds. On the other hand, with lamellar obsidian, quartz, or silver quartzite, which have the lowest bending stress, the fewest workpiece materials could be machined. This shows a correlation between the bending strength of the natural rocks and their suitability as cutting materials.

### 3.3. Tool Loads during Cutting with Natural Rocks

**Figure 7:**
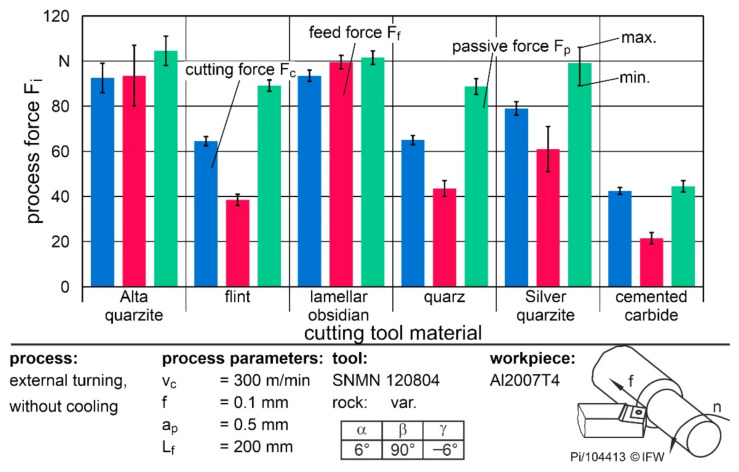
Process forces while turning Al2007T4 with a cutting speed of 300 m/min.

To assess the possibility of rocks as potential cutting materials more precisely, the occurring cutting forces were compared and the significant influencing variables were determined. The forces were compared when machining Al2007T4, since no breakouts occurred regardless of rock type and process parameters. First, the cutting, feed, and passive forces of the five rocks were compared at a feed rate of 0.1 mm and a cutting speed of v_c_ = 300 m/min. Figure 7 shows the mean process forces over a feed path of 200 mm. In addition to the respective forces of the natural rocks, F_c_ and F_f_ during machining with cemented carbide are shown.

The highest cutting force of 95 N occurred when using Alta quartzite and lamellar obsidian. When silver quartzite was used, the F_c_ fell to 80 N, and with flint or quartz it was only approximately 62 N. Furthermore, it was noticeable that, with Alta quartzite and lamellar obsidian, the feed force was equal to the cutting force. Consequently, increased adhesions and built-up edges must be present during the process to increase the force component in the feed direction accordingly. When using the other rocks, the feed force F_f_ was about two-thirds of the cutting force. Overall, the cutting forces of the rocks were between 50% and 120% higher than those of the cemented carbide. The reasons for this are the significantly smaller cutting edge rounding and the low surface roughness around the cutting edge and the flank face surface of Ra = 0.5 µm of the cemented carbide tools. As a result, material adhesion was reduced, even at a low cutting speed, for aluminum machining, and the process forces were correspondingly lower. The same reasons led to the low forces with flint, which has the smallest rounding and lowest roughness after cemented carbide. In contrast, the large roundings of the Alta quartzite and lamellar obsidian, in combination with the high asymmetry towards the flank face, led to the high feed forces. Accordingly, cutting edge preparation after grinding should be considered in future investigations to reduce chipping while providing comparable geometries.

**Figure 8:**
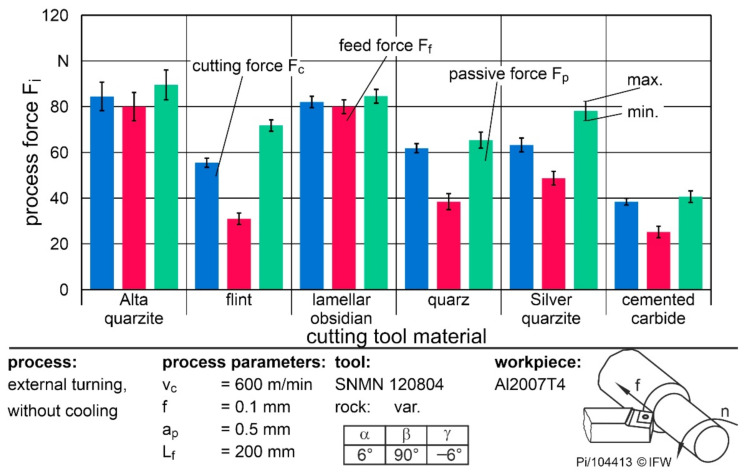
Process forces while turning Al2007T4 with a cutting speed of 600 m/min.

Figure 8 shows the process forces of the cutting tool materials at the same feed rate but at a higher cutting speed of v_c_ = 600 m/min. The doubling of the cutting speed did not lead to any significant reduction in the cutting or feed force when using cemented carbide. Thus, the range of thermal softening of the material had not yet been reached. Nevertheless, the effective forces were reduced by up to 17% when using natural rocks. This was due to the lower adhesion, resulting in a lower built-up edge. This reduction of forces was the reason for the better usability of natural rocks at higher cutting speeds. A higher thermal load and a corresponding reduction of properties thus do not seem to be as critical as the purely mechanical load. One explanation would be the property that natural rocks are good insulators and absorb only a small portion of the cutting heat. This results in a higher thermal softening of the workpiece material. The other effects regarding the ratio of cutting and feed force behaved analogously to the lower cutting speed.

**Figure 9:**
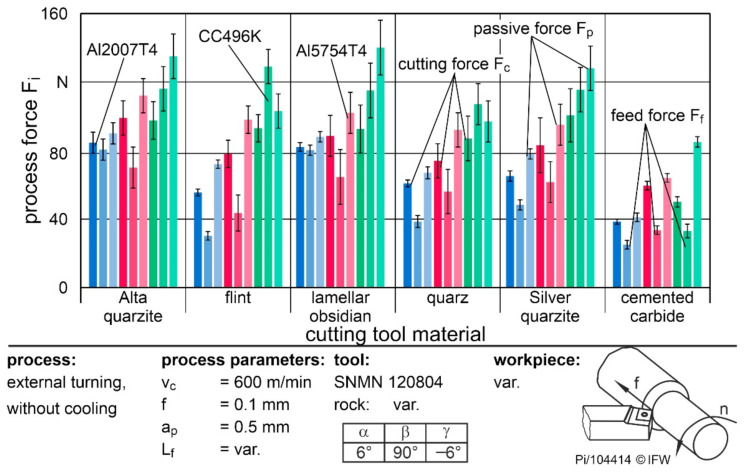
Influence of the materials on the process forces of different natural rocks.

To evaluate the operational behavior for different materials, Figure 9 shows the process forces when machining Al2007T4, Al5754T4, and CC496K with a feed rate of f = 0.1 mm and a cutting speed of v_c_ = 600 m/min.

When machining CC496K with natural rocks, the feed force was roughly 15% higher than the cutting force. Although the tensile strength and hardness of the copper materials were lower than those of the aluminum materials, the flow stress was higher. As a result, the process forces increased during machining. The strong material accumulations were caused by the high springback in the contact area, as a result of the high Young’s modulus of the copper alloy, in combination with the flank face roughness. Accordingly, the increase in the feed and passive forces were the cause of the poor suitability of rocks for copper machining. Since F_f_ acts normally in the direction of the flank face and, especially in the case of quartzites, the cutting wedge is then loaded in such a way that the microtextures are unfavorably aligned to the direction of loading, the wear was additionally increased. Furthermore, natural rocks are particularly susceptible to shear stresses [6].

If the two aluminum alloys are compared, the cutting and feed forces when machining Al5754T4 were about 25% higher than those of Al2007T4 when using flint, quartz or silver quartzite. This was due to the higher material adhesion of the softer workpiece, which in turn led to higher cutting forces that exceeded the strength of the natural rocks. However, if Alta quartzite or lamellar obsidian were used, only the cutting force increased. Nevertheless, the influence of cutting edge microgeometry and surface roughness was noticeable in the material comparison. Thus, in future work, these variables should be optimized during manufacturing. In combination with adapted process parameters for Al2007T4 or the machining of non-adhesive materials such as plastics, natural rocks, especially flint and quartz, are an alternative cutting material.

## 4. Conclusions

Based on the investigations carried out, the range of applications for rocks as an alternative cutting material can be narrowed down further. The following results can be summarized:

- Cutting edge roughness and cutting edge microgeometry after grinding vary depending on the rock properties. The resulting cutting edge roundings are up to four times higher than for a conventional cemented carbide insert.
- Due to the influence of cutting edge microgeometry on the operational behavior of cutting inserts, the application of cutting edge preparation steps after grinding must be considered to achieve higher cutting edge quality.
- Flint and quartz are well suited for machining low-adhesion materials with high cutting speeds due to their hardness and bending strength.
- The influences of feed rate and cutting speed on the cutting forces show the same tendency as conventional cutting materials.
- The large cutting edge microgeometry and surface roughness of the rocks lead to strong material accumulations and high process forces when machining adhesive materials.

In future work, the focus should be on the use of flint as a cutting material when machining aluminum at high cutting speeds. Due to the rock properties, high cutting speeds do not lead to thermal stresses. In conclusion, crack propagation due to thermal shocks can also be excluded, which means that machining can be carried out in the future using coolants. At the same time, the grinding process should be optimized and a combination with a cutting edge preparation should be considered to keep the cutting edge microgeometry and surface roughness as low as possible.

## Figures and Tables

**Table 1:** Properties of the workpiece materials.

Workpiece	Tensile Strength [MPa]	Young’s Modulus [GPa]	Yield Strength [MPa]	Hardness [GPa]	Density [g/cm^3^]	Thermal Conductivity [W/m·K]
Al2007T4	340	73	200	0.98	2.85	145
Al5754T4	230	70	80	0.52	2.67	150
CC496K	200	82	90	0.67	9.20	59
CW614N	360	96	350	0.93	8.46	113

**Table 2:** Cutting edge geometry of the used inserts.

Rock	Cutting Edge Segment on Flank Face S_α_ [µm]	Cutting Edge Segment on Rake Face S_γ_ [µm]	$\mathrm{Average}\;\mathrm{Cutting}-\mathrm{Edge}\;\mathrm{Rounding}\;\bar{S}\;$ [µm]
Alta quartzite	30.9 ± 2.1	49.6 ± 6.3	40.2 ± 3.8
flint	23.3 ± 5.5	28.0 ± 11.8	25.6 ± 8.5
lamellar obsidian	22.7 ± 5.6	36.7 ± 11.3	29.7 ± 8.0
quartz	31.7 ± 12.1	39.3 ± 14.4	35.5 ± 12.9
silver quartzite	33.4 ± 2.5	45.6 ± 11.1	39.5 ± 6.4
cemented carbide	9.4 ± 1.8	11.0 ± 2.6	10.2 ± 2.2

## Data Availability

The data presented in this study are available on request from the corresponding author.

## References

[1] Furberg A., Arvidsson R., Molander S. (2019). Enviromental life cycle assessment of cemented carbide (WC-Co) production. J. Clean. Prod..

[2] Werner A.B.T., Sinclair W.D., Amey E.B. (2014). International Strategic Mineral Issues Summary Report—Tungsten.

[3] Gislev M., Grohol M. (2018). Report on Critical Raw Materials and the Circular Economy.

[4] European Union Project (2017). Flintstone 2020 Next Generation of Superhard Non-CRM Materials and Solutions in Tooling.

[5] Hrechuk A., Johansson D., Bushlya V., Devin L., Ståhl J.-E. (2018). Application of Colding tool life equation on the drilling fiber reinforcement polymers. Procedia Manuf..

[6] Denkena B., Breidenstein B., Krödel A., Bergmann B., Picker T., Wolters P. (2021). Suitability of natural rocks as materials for cutting tools. SN Appl. Sci..

[7] Wolters P., Picker T., Breidenstein B., Krödel A., Denkena B., Behrens B.A., Brosius A., Drossel W.G., Hintze W., Ihlenfeldt S., Nyhuis P. (2021). Application of natural rocks in cutting aluminum. Production at the Leading Edge of Technology. WGP 2021.

[8] Denkena B., Boehnke D., Bockhorst J. (2019). Thin tools for the high speed cutting of granite. Int. J. Abras. Technol..

[9] Almasi S.N., Bagherpour R., Mikaeil R., Ozcelik Y. (2017). Analysis of bead wear in diamond wire sawing considering the rock properties and production rate. Bull. Eng. Geol. Environ..

[10] Miedema S.A. (2014). The Delft Sand, Clay & Rock Cutting Model.

[11] Richard T., Dagrain F., Poyol E., Detournay E. (2012). Rock strength determination from scratch tests. Eng. Geol..

[12] Garner N. (1967). Cutting Action of a Single Diamond Under Simulated Borehole Conditions. J. Pet. Technol..

[13] Nishimatsu Y. (1972). The mechanics of rock cutting. Int. J. Rock Mech. Min. Sci. Geomech. Abstr..

[14] Johansson E. (2011). Technological Properties of Rock Aggregates. Ph.D. Thesis.

[15] Meng F., Wong L.N.Y., Zhou H. (2021). Rock brittleness indices and their applications to different fields of rock engineering: A review. J. Rock Mech. Geotech. Eng..

[16] Denkena B., Grove T., Bremer I., Behrens L. (2016). Design of bronze-bonded grinding wheel properties. CIRP Ann..

[17] Denkena B., Breidenstein B., Bergmann B., Wolters P. (2022). Investigation of the material separation behaviour of rocks using scratch tests for the design of tool grinding processes. Springer Nat. Appl. Sci..

[18] Denkena B., Biermann D. (2014). Cutting edge geometries. CIRP Ann.-Manuf. Technol..

[19] Bergmann B. (2017). Grundlagen zur Auslegung von Schneidkantenverrundungen. Ph.D. Thesis.

[20] Bergmann B., Denkena B., Grove T., Picker T. (2019). Chip formation of rounded cutting edges. Int. J. Precis. Eng. Manuf..

